# Identification of an Immune-Related Prognostic Signature Associated With Immune Infiltration in Melanoma

**DOI:** 10.3389/fgene.2020.01002

**Published:** 2020-08-28

**Authors:** Nian Liu, Zijian Liu, Xinxin Liu, Xiaoru Duan, Yuqiong Huang, Zilin Jin, Yi Niu, Liling Zhang, Hongxiang Chen

**Affiliations:** ^1^Department of Dermatology, Union Hospital, Tongji Medical College, Huazhong University of Science and Technology, Wuhan, China; ^2^Cancer Center, Union Hospital, Tongji Medical College, Huazhong University of Science and Technology, Wuhan, China; ^3^Department of Gastrointestinal Surgery, Union Hospital, Tongji Medical College, Huazhong University of Science and Technology, Wuhan, China; ^4^Department of Dermatology, The 6th Affifiliated Hospital of Shenzhen University Health Science Center, Shenzhen, China; ^5^Department of Dermatology, Union Shenzhen Hospital, Huazhong University of Science and Technology, Shenzhen, China

**Keywords:** melanoma, IRGs, prognostic signature, immune cells infiltration, TMB, immunotherapy

## Abstract

Melanoma is the leading cause of cancer-related death among skin tumors, with an increasing incidence worldwide. Few studies have effectively investigated the significance of an immune-related gene (IRG) signature for melanoma prognosis. Here, we constructed an IRGs prognostic signature using bioinformatics methods and evaluated and validated its predictive capability. Then, immune cell infiltration and tumor mutation burden (TMB) landscapes associated with this signature in melanoma were analyzed comprehensively. With the 10-IRG prognostic signature, melanoma patients in the low-risk group showed better survival with distinct features of high immune cell infiltration and TMB. Importantly, melanoma patients in this subgroup were significantly responsive to MAGE-A3 in the validation cohort. This immune-related prognostic signature is thus a reliable tool to predict melanoma prognosis; as the underlying mechanism of this signature is associated with immune infiltration and mutation burden, it might reflect the benefit of immunotherapy to patients.

## Introduction

Melanoma is the most fatal form of skin tumors and is malignantly transformed from melanocytes (Jackett and Scolyer, 2019). Despite awareness that melanoma progression is primarily caused by complex interactions between environmental and host risk factors (Schadendorf et al., 2018), there were approximately 96,000 new cases and 9,000 deaths of melanoma worldwide in 2018 (Siegel et al., 2018). It is one of the most aggressive cancers that cause more than 75% of skin cancer-related deaths (Gershenwald and Guy, 2015). Although standard surgical resection has improved the prognosis of melanoma patients at localized stage, a large subset of melanoma patients diagnosed with advanced or metastatic melanoma, remains poorly treated.

Due to the complexity of cancer immune response, the contexture and components of immune infiltrates are considerably heterogeneous among each patient and cancer type (Angell and Galon, 2013). Increasing evidence demonstrates that melanoma initiation, evolution, and metastasis are closely related to the tumor microenvironment (TME). In fact, the density of immune contexture has demonstrated a clear correlation with responses to immunotherapy or patients’ clinical outcomes including melanoma (Mann et al., 2013), ovarian clear cell carcinoma (Tan et al., 2019), and bladder cancer (Song et al., 2019). However, the limitations of immune-checkpoint inhibitors (ICIs) in metastatic melanoma require a deep understanding of the definitive mechanisms within the tumor microenvironment and a more reliable biomarker to predict patient prognosis or response to ICIs.

Currently, genetic studies suggest that melanoma is one of the highest mutated malignancies (Alexandrov et al., 2013), of which two prominent mutational events, BARF and NRAS, are observed in approximately 40–60% (Flaherty et al., 2010) and 15–20% (Mandala et al., 2014) of all melanoma cases, respectively. Clinical implications have been observed between mutational burden and susceptibility to immune treatment in the field of oncology (Rizvi et al., 2015; Hugo et al., 2016).

Melanoma, serves as an immunogenic malignancy, developed on the background of these cellular mechanisms including complex interaction among immunomodulatory molecules and high tumor mutational burden (TMB). Given the circumstance that a higher mutation pattern is not always equal to an immunologically hot phenotype (McGranahan et al., 2016), an improved prognostic signature that simultaneously considers the TME and TMB is urgently needed.

In the present study, we downloaded gene expression profiles from The Cancer Genome Atlas (TCGA) as a training cohort and those from the Gene Expression Omnibus (GEO) database as validation cohorts. Based on an immune profile, we used two computational algorithms to screen characteristic genes for further constructing an immune signature with predictive power. We then highlighted the prognostic value and independent role of the resulting 10-gene immune-related model. We also estimated the TME infiltration and mutation pattern in patients from high- and low-risk groups. As a result, we established a robust prognostic biomarker with significantly different TME infiltration and TMB patterns, which can be a potential tool for immunotherapy prediction.

## Materials and Methods

### Screening DE IRGs Between Primary and Metastatic Melanoma

Using the list of immune genes downloaded from the ImmPort website (Bhattacharya et al., 2018)^1^, we screened their expression data in TCGA SKCM data matrix profile during the development of primary and metastatic melanoma. A total of 435 melanoma samples were included in our analysis and the criterion and data processing could be found in our previous study (Liu et al., 2019). Based on |log_2_FC| >1 and false discovery rate (FDR) <0.01, differentially expressed immune-related genes (DE IRGs) were obtained using the edge R package. Subsequently, two feature selection methods, the Least Absolute Shrinkage and Selector Operation (LASSO) (Tibshirani, 1996) and Support Vector Machine-Recursive Feature Elimination (SVM-RFE) (Huang et al., 2014), were applied for in-sample cross-validation and reducing the scope of candidate genes for patients with melanoma. Finally, genes from either the LASSO or SVM-RFE algorithms were subsumed in further analysis.

### Definition of an Immune-Related Prognostic Model

The feature genes were analyzed using univariate and multivariate Cox proportional hazards regression analysis to establish a prognostic predictive model. A risk score formula was constructed as described in our previous study (Liu et al., 2019). To validate the prognostic capability of the model in TCGA set, melanoma patients were divided into high- or low-risk groups determined by the median score as the threshold. Time-dependent receiver operating characteristic (ROC) curve analyses were utilized to evaluate the accuracy and efficiency of the prognostic model using the “survival ROC” package of R. *P* < 0.05 was considered statistically significant. The Kaplan-Meier method was used to compare significant differences in overall survival (OS) between different subgroups. Correlations between the risk score and clinical features of melanoma patients were analyzed using the Chi-square test.

### Development of a Predictive Nomogram

Univariate Cox regression analysis was performed to identify the independent prognostic factors. The clinical characteristics reaching the statistical difference with *P* < 0.05 in univariate analysis could be selected for further nomogram construction. Predictive nomograms were constructed using logistic and Cox regression analysis, respectively. Moreover, calibration curves for 5- and 10-year OS were used to assess the prognostic accuracy of the nomogram. The observed and predicted outcomes of the nomogram were presented in the calibrate curve and the 45° line represented the ideal prediction. The nomograms and calibration curves were constructed using the “rms” package in R.

### Risk-Related Genes Analysis

To explore the biological implications of the immune-related prognostic signature, the melanoma patients were divided into low- and high-risk groups to identify the differentially expressed genes (DEGs) associated with the risk patterns mentioned above. DEGs among these groups were determined using the R package “limma,” with |log_2_FC| >1 and FDR <0.01 regarded as significance criteria.

Gene ontology (GO) enrichment analysis within risk groups was performed to gain insight into the biological functions of prognostic immune-related genes using the Database for Annotation, Visualization, and Integrated Discovery (DAVID) (Huang et al., 2009), with a threshold of *P* < 0.05. Furthermore, significantly enriched GO terms in biological process (BP) were visualized using R packages. Gene set variation analysis (GSVA) (Hänzelmann et al., 2013) was conducted between high- and low-risk groups using the GSVA_1.30.0 package of R.

### Estimation of Tumor Infiltrating Immune Cells

To investigate the immune infiltration landscape between different subgroups, single-sample gene set enrichment analysis (ssGSEA) was used to quantify the infiltration levels of 24 immune cell types, according to the expression levels of the immune cell-specific marker genes described by Bindea et al. (2013). The ssGSEA ranked the genes by their absolute expression in each sample and grouped the different immune cell infiltration patterns based on the R package “gsva.”

Similarly, cell type identification by estimating the relative subsets of RNA transcripts (CIBERSORT)^2^, a deconvolution algorithm, was developed to determine the relative fraction of 22 immune cell types in melanoma tissues (Newman et al., 2015). The transcriptional profiles obtained from TCGA database were prepared in accordance with the accepted format of CIBERSORT and LM22, a gene signature matrix that defines 22 immune cell subtypes, and was used as the signature gene file. CIBERSORT was run with 1,000 permutations and a threshold <0.05 as recommended.

Based on the gene expression signature, “Estimation of STromal and Immune cells in Malignant Tumors using Expression data” (ESTIMATE) was used to assess the estimate score, stromal score, and immune score using the “estimate” package for tumor samples (Yoshihara et al., 2013)^3^.

### Profiles of TMB and Correlation Analysis

The mutation data for melanoma patients were downloaded from TCGA data portal^4^. For each patient, MutSigCV_v1.41^5^ was used to identify the significant mutated genes (*P* < 0.05) across the two classes currently identified with the risk score (Lawrence et al., 2013). The mutation landscape oncoprint was drawn using R package “ComplexHeatmap.”

### Validation Datasets

The robustness of the prognostic model was validated in GSE19234, GSE22153, and GSE35640 based on the GPL570, GPL6102, and GPL570 platforms from the GEO database (Clough and Barrett, 2016)^6^, respectively.

### Availability of Data and Materials

All data used in this study were obtained from the TCGA database: https://portal.gdc.cancer.gov/and GEO database: https://www.ncbi.nlm.nih.gov/geo/.

### Statistical Analysis

All statistical analyses were executed in R version 3.5.2. *P* < 0.05 were considered statistically significant unless otherwise mentioned.

## Results

### Screening Candidate DE IRGs

**FIGURE 1:**
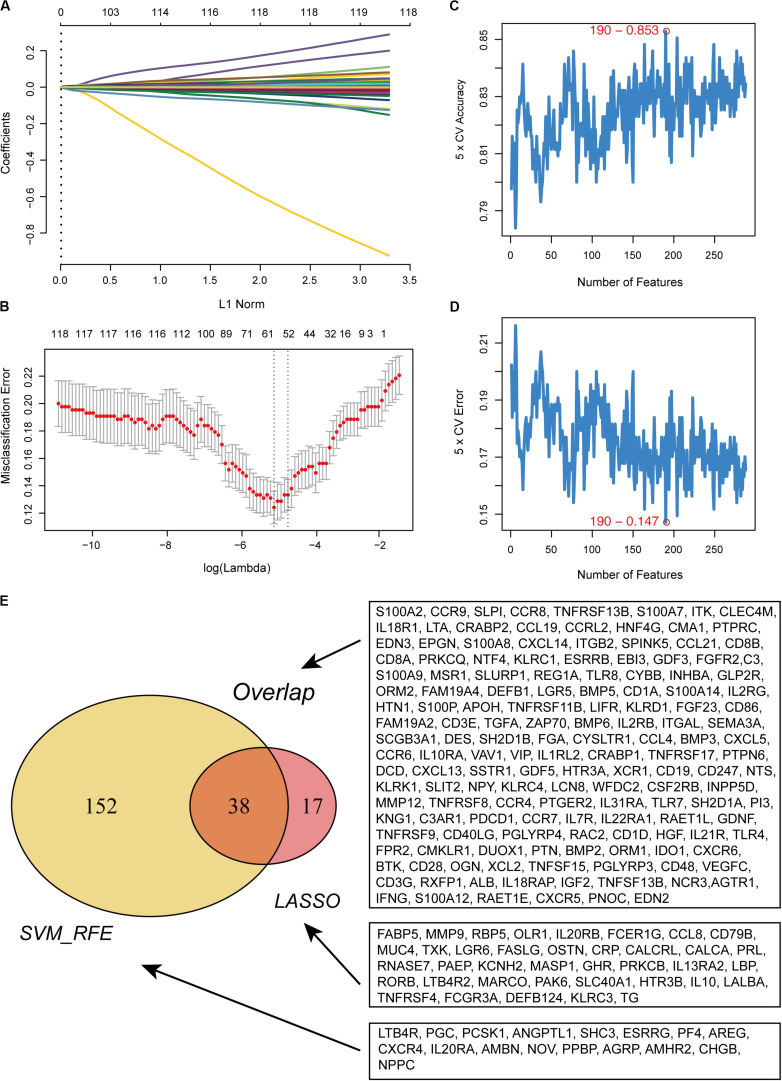
Recognition of candidate DE IRGs. **(A)** 55 DE IRGs were selected on LASSO coefficient profiles. The colored curves correspond to DE IRGs; horizontal axis represents the L1 Norm; vertical lines show the values of coefficients. **(B)** Feature selection used ten-times cross-validation to prevent overfitting and a confidence interval was obtained for optimal parameters. Red points represent log (lambda) values and two gray vertical lines represent the confidence intervals. **(C)** Feature selection based on the fivefold CV accuracy rate via the SVM-RFE algorithm. **(D)** Feature selection based on the fivefold CV error rate via the SVM-RFE algorithm. **(E)** Venn diagram for candidate DE IRGs.

In total, 288 DE IRGs were screened out with the cutoff criteria of |log_2_FC| >1 and FDR <0.01, including 221 upregulated and 67 downregulated genes. For further validation and selection of DE IRGs with significantly characteristic values to classify primary melanoma (PM) and metastatic melanoma (MM), the LASSO algorithm was used to identify a set of 190 DEGs (Figures 1A,B) and the SVM-RFE algorithm was used to select a set of 55 DEGs (Figures 1C,D). Finally, after combing the LASSO and SVM-RFE algorithms, the 207 most representative DEGs were identified, with 38 genes selected simultaneously by the two algorithms (Figure 1E).

### Construction of a Prognostic Immune Gene Signature

In this study, each DE IRG was first submitted for univariate Cox proportional hazards regression with the criteria of a *P* < 0.05 (Supplementary Figure S1A). Then, multivariate Cox regression analysis was performed to develop the prognostic model and the Concordance index was 0.7, which indicated the high predictive accuracy of the signature for survival (*P* < 0.05; Table 1 and Supplementary Figure S1B). The prognostic risk score was calculated as follows: (0.182 ^∗^ S100A12) + (0.390 ^∗^ CCRL2) + (−0.587 ^∗^ CD86) + (−0.2 90 ^∗^ IL21R) + (0.199 ^∗^ CCR4) + (0.375 ^∗^ FCGR3A) + (−0.350 ^∗^ KLRD1) + (−0.147 ^∗^ IL18RAP) + (0.460 ^∗^ IL2RB) + (−0.2 11 ^∗^ CCL8).

**TABLE 1:** Prognostic value of the 10 prognostic IRGs investigated by multivariate Cox regression analysis.

IRGs	coef	exp (coef)	se (coef)	z	*P*-value
S100A12	0.182	1.200	0.042	4.360	1.30E−05
CCRL2	0.390	1.477	0.112	3.483	0.000496
CD86	−0.587	0.556	0.160	−3.674	0.0002383
IL21R	−0.290	0.748	0.087	−3.334	0.0008559
CCR4	0.199	1.220	0.070	2.856	0.0042891
FCGR3A	0.375	1.455	0.110	3.413	0.0006419
KLRD1	−0.350	0.705	0.088	−3.983	6.81E−05
IL18RAP	−0.147	0.863	0.070	−2.097	0.0359954
IL2RB	0.460	1.583	0.091	5.060	4.20E−07
CCL8	−0.211	0.810	0.060	−3.493	0.0004771

**FIGURE 2:**
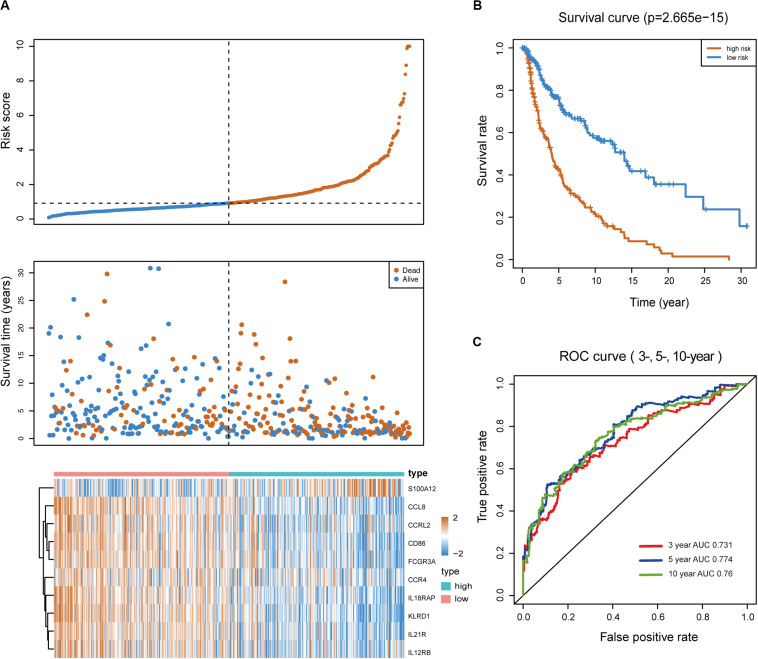
Predictive value of the immune-related signature. **(A)** Risk score distribution, survival status, and expression profiles of the signature. **(B)** Kaplan-Meier survival analysis of the 10-IRG prognostic signature for patients with melanoma. Red line indicates the high-risk group; blue line indicates the low-risk group. **(C)** Time-dependent ROC analysis of the sensitivity and specificity of the risk signature. Red plot represents the 3-year OS rates (AUC = 0.731); blue plot represents the 5-year OS rates (AUC = 0.774); green plot represents the 10-year OS rates (AUC = 0.76).

Melanoma patients were assigned to the high- and low-risk groups based on the risk score model. Distribution of risk score, patients’ survival status, and gene expression profiles associated with the risk score are shown in Figure 2A. Kaplan-Meier analysis demonstrated that melanoma patients with a high-risk score showed dramatically worse prognosis than those with a low-risk score (Figure 2B), and the area under the curve values of the time-dependent ROC curve were 0.731, 0.774, and 0.76 for 3-, 5-, and 10-year survival, indicating the high specificity and sensitivity of the prognostic signature (Figure 2C).

### Stratification Analyses of the 10-Gene Prognostic Signature

Considering the potential impacts of clinical characteristics on the risk score of the prognostic model, a Chi-square test was performed. The risk score exhibited a higher level in event and the American Joint Committee on Cancer-Tumor (AJCC-T) subgroups; there were no significant differences in the risk score, AJCC-Nodes (AJCC-N), AJCC-Metastasis (AJCC-M), Stage, Clark, Primary Tumor, and the BRAF and NRAS subgroups (Supplementary Figure S2A). To assess whether the prognostic classifier was an independent indicator in distinct subgroups, we checked the effect of BRAF and NRAS mutation or wild-type on the prognostic ability for melanoma in TCGA cohort. Kaplan-Meier analysis revealed that patients in the high-risk group were associated with a higher mortality risk in the BRAF^wt^ and NRAS^wt^ or BRAF^mut^ and NRAS^mut^ subgroups (Supplementary Figures S2B–E). Additionally, patients in stages I and II or stages III and IV were divided into two groups with the median value. We found that patients with high risk were classified into the group with a worse prognosis, compared to patients with a low risk (Supplementary Figures S2F,G). These results demonstrated the robust and predictive power of this prognostic model, in which patients high-risk scores had a shorter overall survival than those with low risk scores in each stratum.

### Construction of a Nomogram Model

**FIGURE 3:**
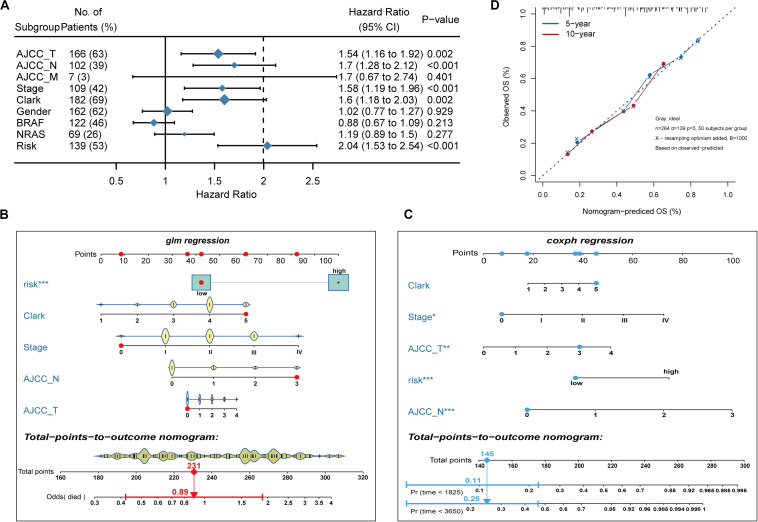
Nomogram model establishment. **(A)** The forest plot of univariate Cox regression analysis was used to show the HR, 95% CI of each variable and *P*-value. **(B)** Nomogram model construction for predicting the probability of melanoma patients with mortality risk odds. **(C)** Nomogram model construction for predicting the probability of melanoma patients with OS rates. **(D)** Nomogram calibration curves for predicting 5- and 10-year OS in melanoma patients. Nomogram-predicted OS is plotted on the x-axis; observed OS is plotted on the y-axis; a plot along the 45° line represents a perfect calibration model.

To develop a clinically relevant quantitative approach for predicting the survival probability of a patient with melanoma, we constructed predictive nomograms. Based on univariate analysis (Figure 3A), we generated two nomograms to predict the death odds of patients using logistic regression and survival rate with Cox regression analysis (Figures 3B,C). The calibration plots for the 5- and 10-year survival showed an optimal agreement between the nomogram-predicted and observed OS (Figure 3D).

### Functional Traits of the Prognostic Signature

**FIGURE 4:**
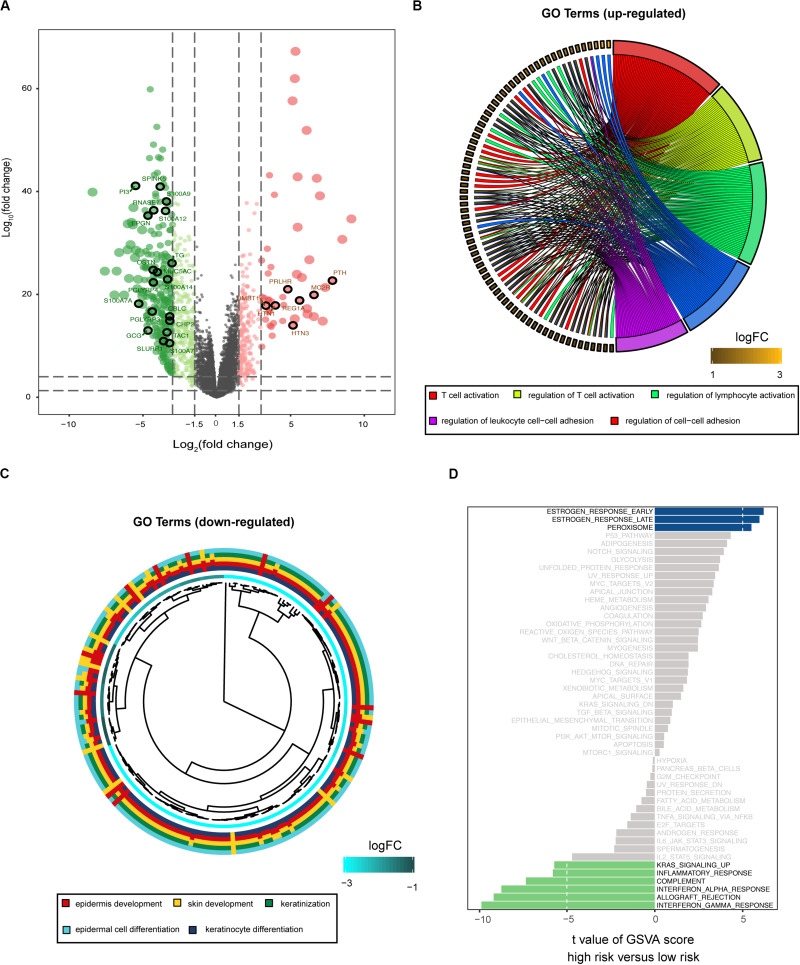
Biological function associated with the risk-related DEGs. **(A)** Volcano plot for DEGs between the high- and low-risk groups. Among these DEGs, immune-related genes are highlighted. GO enrichment analysis for the upregulated **(B)** and downregulated **(C)** gene clusters. **(D)** GSVA demonstrates upregulated immune-related pathways in the low-risk group.

To explore the potential cause of the prognostic signature, we divided the melanoma patients from TCGA database into high- and low-risk groups, based on the median risk score. After edgeR filtering (|log_2_FC| > 1 and FDR < 0.05), we screened out 1,251 DEGs, including 552 upregulated and 699 downregulated genes (Figure 4A). Of these DEGs, 26 genes were immune-related and are highlighted in Figure 4A. GO enrichment analysis revealed that upregulated genes were significantly enriched in multiple pathways including T cell activation, regulation of T cell activation, and regulation of lymphocytes (*P* < 0.05; Figure 4B). Moreover, downregulated genes were significantly enriched in epidermis development, skin development, and keratinization (*P* < 0.05; Figure 4C). GSVA showed that patients with low risk scores exhibited increased expression of proteins associated with the interferon gamma response, allograft rejection, and interferon alpha response (Figure 4D). These findings indicate differences in the immune-related genes and signaling pathways between high- and low-risk groups, which might partly explain the reason for the significant difference in prognosis between the subgroups.

### The Risk Score Was Associated With Immune Cell Infiltration

**FIGURE 5:**
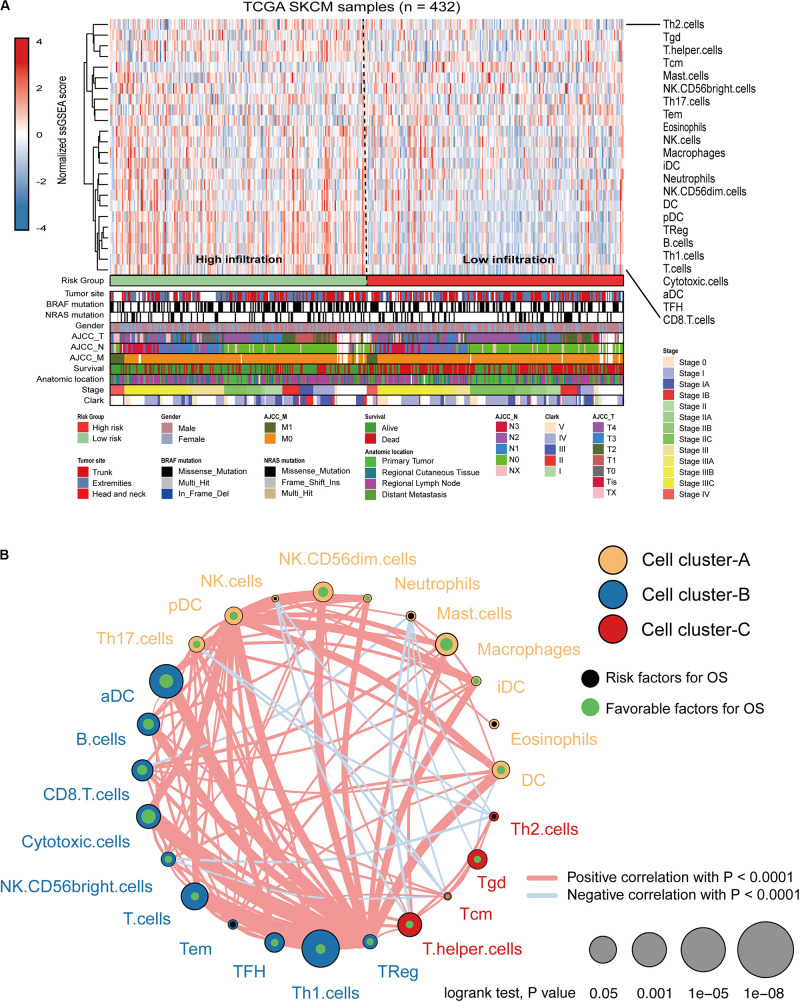
Immune characteristics of melanoma patients estimated by the ssGSEA algorithm. **(A)** The landscape of immune cells using the ssGSEA scores. Tumor site, mutation status of BRAF and NRAS, gender, AJCC-T, AJCC-N, AJCC-M, survival, anatomic location, stage, and Clark status are shown as patient annotations in the lower panel. Two distinct immune infiltration clusters, termed high infiltration and low infiltration, were identified by the risk groups. **(B)** Cellular interaction of the TME immune cell types. Three immune cell clusters were defined as cluster-A, cluster-B, and cluster-C. The thickness of the line between immune cells indicates the strength of the correlation.

Immune cell infiltration status was assessed by applying the ssGSEA approach to the melanoma transcriptomes. Twenty-four immune-related terms were incorporated to deconvolve the abundance of diverse immune cell types in melanoma. The whole cohort was clustered into two clusters in terms of immune infiltration by applying the IRGs signature (Figure 5A) and the relative immune score in ssGSEA is shown in Supplementary Figure S3A. Subsequently, a TME cells network was constructed with three main clusters depicting a comprehensive landscape of tumor-immune cell interactions and cell lineage, and their effects on the OS of melanoma patients (Supplementary Figure S3B and Figure 5B). ssGSEA was used to assess the relative proportions of the 24 immune cells in each CC sample. The abundance of aDC, B cells, CD8 T cells, cytotoxic cells, DC, macrophages, NK CD56^dim^ cells, pDC, T cells, T helper cells, Tcm, TFH, Th1, and Treg cells was low in the 10-IRG signature high-risk group and associated with better OS. The abundance of eosinophils and mast cells was high in the 10-IRG signature high-risk group and associated with poor OS.

**FIGURE 6:**
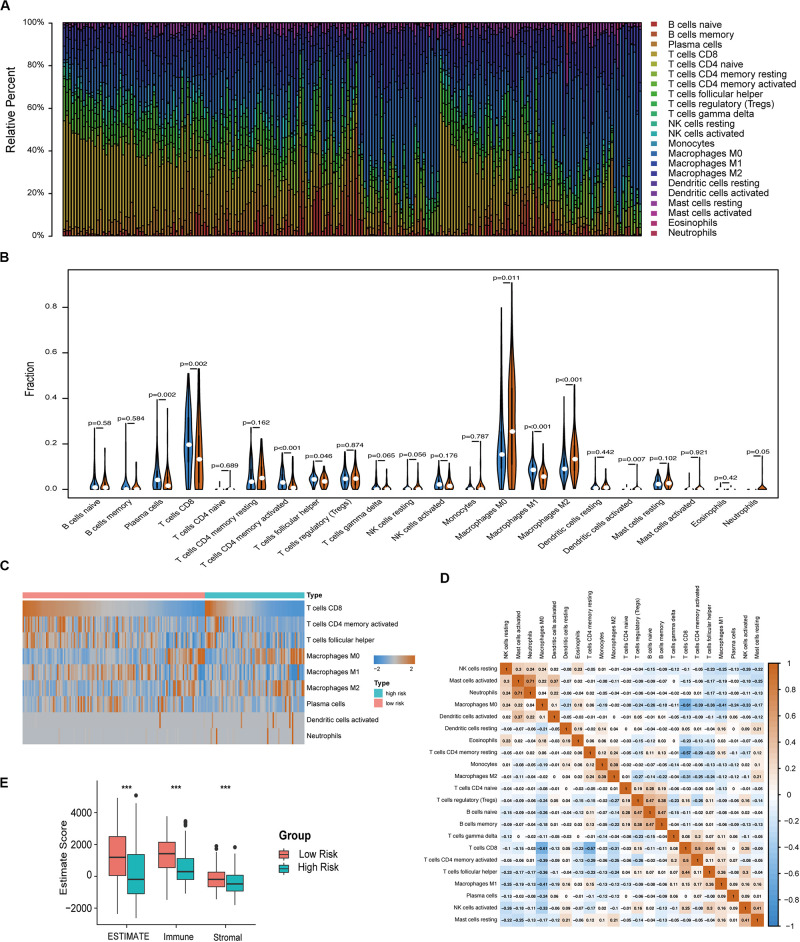
Immune characteristics of melanoma patients estimated by CIBERSORT and ESTIMATE algorithms. **(A)** The distribution of 22 immune cell infiltrations in each melanoma sample. The lengths of the bars indicate the levels of immune cell populations and different colors represent different types of immune cells. **(B)** The association of 22 immune cells infiltration abundances and risk scores. The blue and red violins represent the 10-IRG signature low- and high-risk group, respectively. The white points inside the violin represent median values. **(C)** Heatmap of the tumor infiltrating cells with statistical significance. **(D)** Correlation matrix for all 22 immune cell proportions. A fraction of immune cells was negatively related and is represented in blue, whereas others were positively related and are represented in red. **(E)** Distributions and comparisons of Estimate, stromal, and immune scores among melanoma patients with different prognosis. The red box represents the low-risk group and the green box represents the high-risk group.

The immune infiltration in melanoma tissues between the high- and low-risk groups was then investigated using the CIBERSORT algorithm. The proportion of 22 immune cells in each subgroup is shown in a bar plot (Figure 6A). The results revealed that plasma cells, CD8 T cells, CD4 memory activated T cells, follicular helper T cells, and M1 macrophages were negatively correlated with the risk score (Figures 6B,C) and that M0 macrophages, M2 macrophages, activated DCs, and neutrophils were positively correlated with the risk score (Figures 6B,C). Figure 6D indicated the poor correlation coefficient between 22 immune cells. The population with negative relation included M0 macrophages and CD8 T cells (*r* = −0.61), and CD4 memory resting T cells and CD8 T cells (*r* = −0.57). Further, neutrophils and activated mast cells had a positive relation (*r* = 0.71). After the ESTIMATE algorithm was processed, a higher Estimate score was found in the low-risk group. Similarly, the fraction of immune and stromal cells was associated with the low-risk group (Figure 6E).

### Different Tumor Mutation Burden (TMB) Patterns Between Two Risk Groups

**FIGURE 7:**
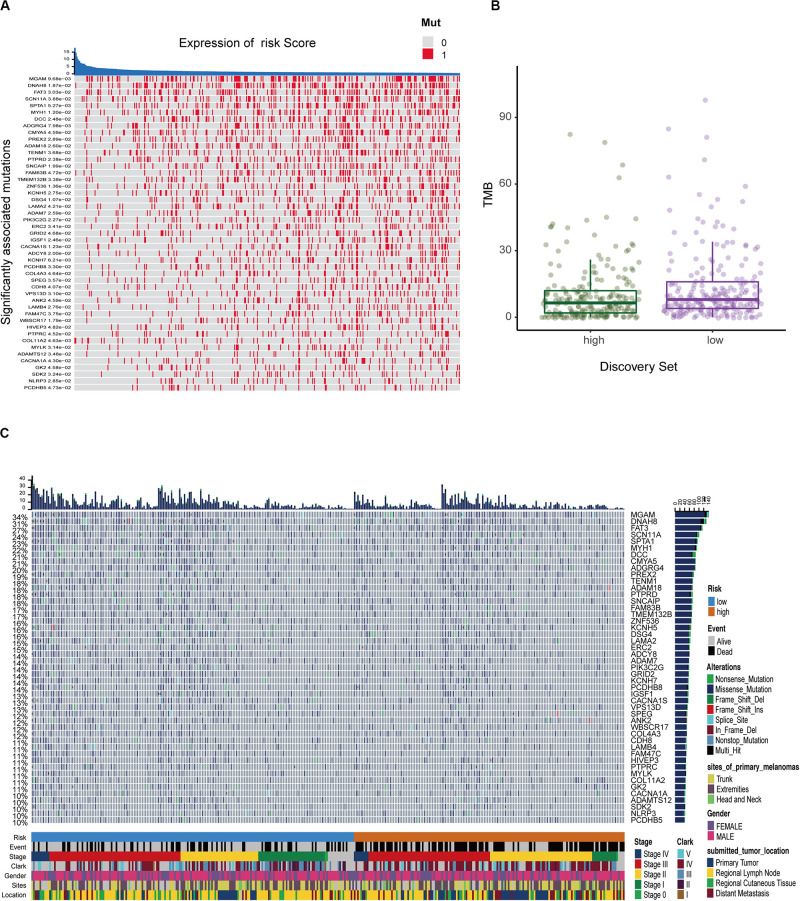
The mutation profile and clinical information of risk subgroups. **(A)** The heatmap shows the correlation between risk score and gene mutations (*P* < 0.05). Red marks the mutations, gray marks the non-mutation. **(B)** Mutation frequency between the high- and low-risk groups. **(C)** Mutational landscape and clinical characteristics of melanoma patients including event, stage, Clark status, gender, sites, and location. The right bar plot shows the mutational frequency of each gene.

We defined and calculated the TMB variable between low- and high-risk groups. We also assessed the correlation between risk score and mutated genes and the mutant rate in these 47 mutants, which were distributed in more than 10% of melanoma samples, and were significantly associated with risk scores at *P* < 0.05 (Figure 7A). The mutational landscape indicated that mutation events occurred more frequently in the low-risk group than in the high group (Figures 7B,C). Moreover, we further analyzed the survival significance of TMB in melanoma and found that lower TMB levels were associated with worse overall survival outcomes (Figure 7C).

### Validation of Signature

To substantiate the stability of the prognostic signature, three external validation cohorts were analyzed. For the external validation cohort 1 and 2, GSE19234 and GSE22155 contained 44 and 57 melanoma patients, respectively. Consistent with previous results, the low risk group had higher levels of immune cell infiltration (Supplementary Figures S4A,S5A). The relative immune score is shown in Supplementary Figures S4B,S5B. As expected, patients in the high-risk group had a significantly increased mortality risk compared with those in the low-risk group (Supplementary Figures S4C,S5C). For the external validation cohort 3, GSE35640 contained 65 patients with MAGE-A3 antigen-specific cancer immunotherapy. Similar analysis showed that the low-risk group had higher levels of immune cell infiltration (Supplementary Figure S6). Furthermore, the prognostic model demonstrated the potential to predict the effect of MAGE-A3 immunotherapy in melanoma patients (Supplementary Figure S6).

## Discussion

The clinical heterogeneity of melanoma suggested that biologically relevant differences might exist within subtypes. The purpose of the study was to identify an immune gene expression signature to predict the prognosis in patients between primary and metastatic melanoma. A flowchart of the analysis procedure for this study was shown in Supplementary Figure S7. According to the DE IRGs obtained in our study, we constructed a 10-IRG prognostic signature independent of previously known clinicopathological factors such as the mutant status. The enriched terms for biological process and immune infiltration landscape revealed that the TME might contribute to tumor progression and a poorer prognosis in melanoma, which was validated in two external datasets. Subsequently, our data demonstrated that most tumors could be categorized as either high TMB with a better prognosis or low TMB with a worse prognosis. This classification of melanoma identified in our study demonstrates that patients with a low risk score had increased immune cell infiltration and TMB, and were more likely to respond to ICIs.

We constructed and validated an immune-related risk signature for melanoma using TCGA and GEO datasets. The signature was composed of 10 DE IRGs with prognostic capability. In this prognostic model, five DE IRGs (S100A12, CCRL2, CCR4, FCGR3A, and IL2RB) were used as risk factors with positive coefficients, whereas the other five genes (CD86, IL21R, KLRD1, IL18RAP, and CCL8) were protective factors with negative coefficients. Furthermore, there were significant differences in survival curves between patients with high and low risk scores. The signature exhibited a firm predicting capability in the training and validation datasets and the high prognostic categorization performance of the immune signature was assuredly due to our stratified analysis strategy. Furthermore, two nomograms were built based on univariate Cox regression coefficients of Risk, Clark status, Stage, AJCC-N, and AJCC-T to evaluate the prognosis predictions of the immune signature. A satisfactory agreement between the observed values and the predicted values for the 5- and 10-years OS, was observed in the calibration curves. These results indicated that this model might be an effective tool for outcome prediction of individualized melanoma patients.

Moreover, we reclassified the microarray data into DEGs according to the median risk score. Functional enrichment analysis indicated that risk-related DEGs were primarily involved in a multitude of immune pathways. We speculated that locoregional immune status might have the potential to improve melanoma prognosis classification. The TME consists of cellular components (immune cells, etc.) as well as non-cellular components (cytokines, etc.). Notably, the complex interplay between tumor cells and their surrounding microenvironment plays a pivotal role during tumor development and has significant effects on the OS and immunotherapeutic efficacy in tumors (Mann et al., 2013; Song et al., 2019; Tan et al., 2019). Here, we investigated the immune landscape between patients in the high-risk and low-risk groups using two bioinformatic methods to infer specific immune cell infiltration. In our analysis, melanoma patients were clustered into two main clusters (high or low immune infiltration). Patients with better prognosis were found in the high infiltration cluster. Furthermore, infiltration of CD103 + CD8 + T cells has been associated with longer survival in patients with melanoma tumors (Edwards et al., 2018). Among these immune cells, the abundance of CD8 T cells was high in the 10-IRG signature low-risk group and was associated with better OS based on the results of the ssGSEA and CIBERSORT algorithms. Based on the results of the ssGSEA, the abundance of immune cells with anti-tumor effects including CD8 T cells, TH1 cells and B cells, was high in low-risk group, though the distribution in high-risk group showed less immune cells, named “cold tumor” (Galon and Bruni, 2018). Subsequently, CIBERSORT was used to assess the relative proportions of 22 immune cells in each melanoma sample. The abundance of immune cells with anti-tumor effects including CD8 T cells, CD4 T cells and M1 macrophages, was high in the low-risk group. However, in high-risk group, the abundance of M2 macrophages required for cancer progression and unactivated M0 macrophages were high, indicating that there was a tendency to transform into M2 macrophages with anti-inflammatory and tumor promoting effects (Mills et al., 2016). And previous studies have been reported that patients with more anti-tumor immune cells infiltration, named “hot tumor,” would have better survival prognosis (Galon and Bruni, 2018). Furthermore, using the ESTIMATE method to evaluate the main non-tumor components in the microenvironment, we found that the low-risk group was profoundly associated with either a higher immune score or a higher stromal score. These results may partially explain the predictive value of this signature.

Due to the prevalence of somatic mutations in the melanoma genome, further mutation analysis was performed to explore the possible mechanisms underlying the signature’s prognostic value. In our model, the risk score was in contrast to the TMB pattern to determine the prognosis of melanoma patients, suggesting that the poor prognosis of the high-risk group may be due to fewer mutant genes in this group. Recently, some studies have indicated that increased tumor mutation loads were associated with survival benefit from both anti-CTLA-4 and anti-PD-1 therapy in multiple malignancies such as melanoma (Hugo et al., 2016), lung cancer (Rizvi et al., 2015), and esophagogastric cancer (Greally et al., 2019). Consistent with these studies, our results showed that melanoma patients in the low-risk group with higher immune infiltration and tumor mutation load might respond well to immunotherapy, though larger studies will be necessary to confirm this finding.

Previous studies have explored whether immune-related biomarkers could be indicators for prognosis or response to immune therapy of melanoma (Cursons et al., 2019; Nie et al., 2019; Poźniak et al., 2019; Yang et al., 2020). Compared with these studies, we used a combination strategy to control the robustness of the predictions of DE IRGs. Based on stratified analysis, we also determined the predictability of the nomogram description model, which fully demonstrated the predictability of the model identified in this study. Poźniak et al. (2019) and Yang et al. (2020) evaluated the abundance of infiltrating immune cells between prognostic immune subgroups. However, our study systematically evaluated the landscape of immune cells in melanoma samples using three bioinformatics tools for cross validation. Notably, our prognostic model demonstrated the compatibility of immune signature with TMB in a multi-omics manner, which should be highlighted during the management of immunotherapeutic combinations. Ultimately, we used this model on its immune profile to evaluate the response to ICIs in melanoma and observed positive results in this study.

Despite these promising results, our study has some limitations. The melanoma samples included in our studies were obtained from available public data; the clinical utility of the model needs to be confirmed in a large-scale of melanoma patients. Though the prognostic value of the immune-related signature was validated well in two GEO cohorts, supplemental basic experiments are still warranted to uncover the biological mechanisms of 10 DE IRGs in the promotion of tumor development. Furthermore, due to limited data, the relevance of the signature to ICI response is not fully understood and requires further research.

## Conclusion

In the current study, we performed a comprehensive evaluation of the prognostic signature generated and validated in our study, which might be a clinically promising tool to classify melanoma patients into subgroups with distinct outcomes, immunophenotypes, mutation patterns, and even their distinct responses to immune therapy. It provides new implications regarding the melanoma immune microenvironment, TMB, and immune-related therapy.

## Data Availability Statement

Publicly available datasets were analyzed in this study. All data used in this study were obtained from the TCGA database: https://portal.gdc.cancer.gov/ and GEO database: https://www.ncbi.nlm.nih.gov/geo/.

## Author Contributions

NL, ZL, and HC designed the experiments. NL, XL, and XD collected the data. NL, ZL, YH, ZJ, and YN analyzed the data. NL and ZL wrote the manuscript. HC and LZ reviewed the manuscript. All authors contributed to the article and approved the submitted version.

## Conflict of Interest

The authors declare that the research was conducted in the absence of any commercial or financial relationships that could be construed as a potential conflict of interest.

## Footnotes

1 http://www.immport.org/

2 https://cibersort.stanford.edu/

3 https://sourceforge.net/projects/estimateproject/

4 https://portal.gdc.cancer.gov/

5 www.broadinstitute.org

6 https://www.ncbi.nlm.nih.gov/geo/

## Abbreviations

AJCC-T: American Joint Committee on Cancer-Tumor
BP: biological process
DEGs: differentially expressed genes
ESTIMATE: Estimation of STromal and Immune cells in Malignant Tumors using Expression data
GEO: Gene Expression Omnibus
GO: Gene ontology
GSVA: Gene set variation analysis
ICIs: immune-checkpoint inhibitors
IRG: immune-related gene
LASSO: Least Absolute Shrinkage and Selector Operation
OS: overall survival
ROC: receiver operating characteristic
ssGSEA: single-sample gene set enrichment analysis
SVM-RFE: Support Vector Machine-Recursive Feature Elimination
TCGA: The Cancer Genome Atlas
TMB: tumor mutation burden
TME: tumor microenvironment.

## References

[B1] Alexandrov L. B. Nik-Zainal S. Wedge D. C. Aparicio S. A. Behjati S. Biankin A. V. (2013). Signatures of mutational processes in human cancer. *Nature* 500 415–421. 23945592 10.1038/nature12477 PMC3776390

[B2] Angell H. Galon J. (2013). From the immune contexture to the immunoscore: the role of prognostic and predictive immune markers in cancer. *Curr. Opin. Immunol.* 25 261–267. 10.1016/j.coi.2013.03.004 23579076

[B3] Bhattacharya S. Dunn P. Thomas C. G. Smith B. Schaefer H. Chen J. (2018). ImmPort, toward repurposing of open access immunological assay data for translational and clinical research. *Sci. Data* 5:180015. 10.1038/sdata.2018.15 PMC5827693 29485622

[B4] Bindea G. Mlecnik B. Tosolini M. Kirilovsky A. Waldner M. Obenauf A. C. (2013). Spatiotemporal dynamics of intratumoral immune cells reveal the immune landscape in human cancer. *Immunity* 39 782–795. 10.1016/j.immuni.2013.10.003 24138885

[B5] Clough E. Barrett T. (2016). The gene expression omnibus database. *Methods Mol. Biol.* 1418 93–110. 10.1007/978-1-4939-3578-9_5 27008011 PMC4944384

[B6] Cursons J. Souza-Fonseca-Guimaraes F. Foroutan M. Anderson A. Hollande F. Hediyeh-Zadeh S. (2019). A gene signature predicting natural killer cell infiltration and improved survival in melanoma patients. *Cancer Immunol. Res.* 7 1162–1174. 10.1158/2326-6066.cir-18-0500 31088844

[B7] Edwards J. Wilmott J. S. Madore J. Gide T. N. Quek C. Tasker A. (2018). CD103+ tumor-resident CD8+ T cells are associated with improved survival in immunotherapy-naïve melanoma patients and expand significantly during anti-PD-1 treatment. *Clin. Cancer Res.* 24 3036–3045. 10.1158/1078-0432.ccr-17-2257 29599411

[B8] Flaherty K. T. Puzanov I. Kim K. B. Ribas A. McArthur G. A. Sosman J. A. (2010). Inhibition of mutated, activated BRAF in metastatic melanoma. *N. Engl. J. Med.* 363 809–819. 20818844 10.1056/NEJMoa1002011 PMC3724529

[B9] Galon J. Bruni D. (2018). Approaches to treat immune hot, altered and cold tumours with combination immunotherapies. *Nat. Rev. Drug Discov.* 18 197–218. 10.1038/s41573-018-0007-y 30610226

[B10] Gershenwald J. E. Guy G. P. Jr. (2015). Stemming the rising incidence of melanoma: calling prevention to action. *J. Natl. Cancer Inst.* 108:djv381. 10.1093/jnci/djv381 PMC6048594 26563358

[B11] Greally M. Chou J. F. Chatila W. K. Margolis M. Capanu M. Hechtman J. F. (2019). Clinical and molecular predictors of response to immune checkpoint inhibitors in patients with advanced esophagogastric cancer. *Clin. Cancer Res.* 25 6160–6169. 10.1158/1078-0432.ccr-18-3603 31337644 PMC6905384

[B12] Hänzelmann S. Castelo R. Guinney J. (2013). GSVA: gene set variation analysis for microarray and RNA-seq data. *BMC Bioinform.* 14:7. 10.1186/1471-2105-14-7 23323831 PMC3618321

[B13] Huang D. W. Sherman B. T. Lempicki R. A. (2009). Systematic and integrative analysis of large gene lists using DAVID bioinformatics resources. *Nat. Protoc.* 4 44–57. 10.1038/nprot.2008.211 19131956

[B14] Huang M. L. Hung Y. H. Lee W. M. Li R. K. Jiang B. R. (2014). SVM-RFE based feature selection and Taguchi parameters optimization for multiclass SVM classifier. *Sci. World J.* 2014:795624. 10.1155/2014/795624 PMC4175386 25295306

[B15] Hugo W. Zaretsky J. M. Sun L. Song C. Moreno B. H. Hu-Lieskovan S. (2016). Genomic and transcriptomic features of response to anti-PD-1 therapy in metastatic melanoma. *Cell* 165 35–44. 10.1016/j.cell.2016.02.065 26997480 PMC4808437

[B16] Jackett L. A. Scolyer R. A. (2019). A review of key biological and molecular events underpinning transformation of melanocytes to primary and metastatic melanoma. *Cancers* 11:E2041. 10.3390/cancers11122041 PMC6966527 31861163

[B17] Lawrence M. S. Stojanov P. Polak P. Kryukov G. V. Cibulskis K. Sivachenko A. (2013). Mutational heterogeneity in cancer and the search for new cancer-associated genes. *Nature* 499 214–218. 23770567 10.1038/nature12213 PMC3919509

[B18] Liu N. Liu Z. Liu X. Chen H. (2019). Comprehensive analysis of a competing endogenous RNA network identifies seven-lncRNA signature as a prognostic biomarker for melanoma. *Front. Oncol.* 9:935. 10.3389/fonc.2019.00935 31649871 PMC6794712

[B19] Liu N. Liu Z. Liu X. Duan X. Huang Y. Jin Z. (2020). Identification of an immune-related prognostic signature associated with immune infiltration in melanoma. *Research Square* [Preprint], 10.21203/rs.3.rs-21119/v1 PMC7484056 33005180

[B20] Mandala M. Merelli B. Massi D. (2014). Nras in melanoma: targeting the undruggable target. *Crit. Rev. Oncol. Hematol.* 92 107–122. 10.1016/j.critrevonc.2014.05.005 24985059

[B21] Mann G. J. Pupo G. M. Campain A. E. Carter C. D. Schramm S. J. Pianova S. (2013). BRAF mutation, NRAS mutation, and the absence of an immune-related expressed gene profile predict poor outcome in patients with stage III melanoma. *J. Invest. Dermatol.* 133 509–517. 10.1038/jid.2012.283 22931913

[B22] McGranahan N. Furness A. J. Rosenthal R. Ramskov S. Lyngaa R. Saini S. K. (2016). Clonal neoantigens elicit T cell immunoreactivity and sensitivity to immune checkpoint blockade. *Science* 351 1463–1469. 26940869 10.1126/science.aaf1490 PMC4984254

[B23] Mills C. D. Lenz L. L. Harris R. A. (2016). A breakthrough: macrophage-directed cancer immunotherapy. *Cancer Res.* 76 513–516. 10.1158/0008-5472.can-15-1737 26772756 PMC4738030

[B24] Newman A. M. Liu C. L. Green M. R. Gentles A. J. Feng W. Xu Y. (2015). Robust enumeration of cell subsets from tissue expression profiles. *Nat. Methods* 12 453–457. 10.1038/nmeth.3337 25822800 PMC4739640

[B25] Nie R. C. Yuan S. Q. Wang Y. Chen Y. B. Cai Y. Y. Chen S. (2019). Robust immunoscore model to predict the response to anti-PD1 therapy in melanoma. *Aging* 11 11576–11590. 10.18632/aging.102556 31796647 PMC6932919

[B26] Poźniak J. Nsengimana J. Laye J. P. O’Shea S. J. Diaz J. M. S. Droop A. P. (2019). Genetic and environmental determinants of immune response to cutaneous melanoma. *Cancer Res.* 79 2684–2696. 10.1158/0008-5472.can-18-2864 30773503 PMC6544535

[B27] Rizvi N. A. Hellmann M. D. Snyder A. Kvistborg P. Makarov V. Havel J. J. (2015). Mutational landscape determines sensitivity to PD-1 blockade in non-small cell lung cancer. *Science* 348 124–128. 25765070 10.1126/science.aaa1348 PMC4993154

[B28] Schadendorf D. van Akkooi A. C. J. Berking C. Griewank K. G. Gutzmer R. Hauschild A. (2018). Melanoma. *Lancet* 392 971–984. 30238891 10.1016/S0140-6736(18)31559-9

[B29] Siegel R. L. Miller K. D. Jemal A. (2018). Cancer statistics. *CA Cancer J. Clin.* 68 7–30. 29313949 10.3322/caac.21442

[B30] Song B. N. Kim S. K. Mun J. Y. Choi Y. D. Leem S. H. Chu I. S. (2019). Identification of an immunotherapy-responsive molecular subtype of bladder cancer. *eBio Med.* 50 238–245. 10.1016/j.ebiom.2019.10.058 31735557 PMC6921227

[B31] Tan T. Z. Ye J. Yee C. V. Lim D. Ngoi N. Y. L. Tan D. S. P. (2019). Analysis of gene expression signatures identifies prognostic and functionally distinct ovarian clear cell carcinoma subtypes. *eBio Med.* 50 203–210. 10.1016/j.ebiom.2019.11.017 31761620 PMC6921362

[B32] Tibshirani R. (1996). Regression shrinkage and selection via the LASSO. *J. R. Stat. Soc. Ser. B* 58 267–288. 10.1111/j.2517-6161.1996.tb02080.x

[B33] Yang S. Liu T. Nan H. Wanåg Y. Chen H. Zhang X. (2020). Comprehensive analysis of prognostic immune-related genes in the tumor microenvironment of cutaneous melanoma. *J. Cell. Physiol.* 235 1025–1035. 10.1002/jcp.29018 31240705

[B34] Yoshihara K. Shahmoradgoli M. Martínez E. Vegesna R. Kim H. Torres-Garcia W. (2013). Inferring tumour purity and stromal and immune cell admixture from expression data. *Nat. Commun.* 4 1–11. 10.1038/ncomms3612 PMC3826632 24113773

