# Supplementary material for: Identification of an Immune-Related Prognostic Signature Associated With Immune Infiltration in Melanoma
Source: Front Genet. 2020 Aug 28;11:1002. doi: 10.3389/fgene.2020.01002 (PMC7484056; doi:10.3389/fgene.2020.01002)

Supplementary Material

## Supplementary Figures


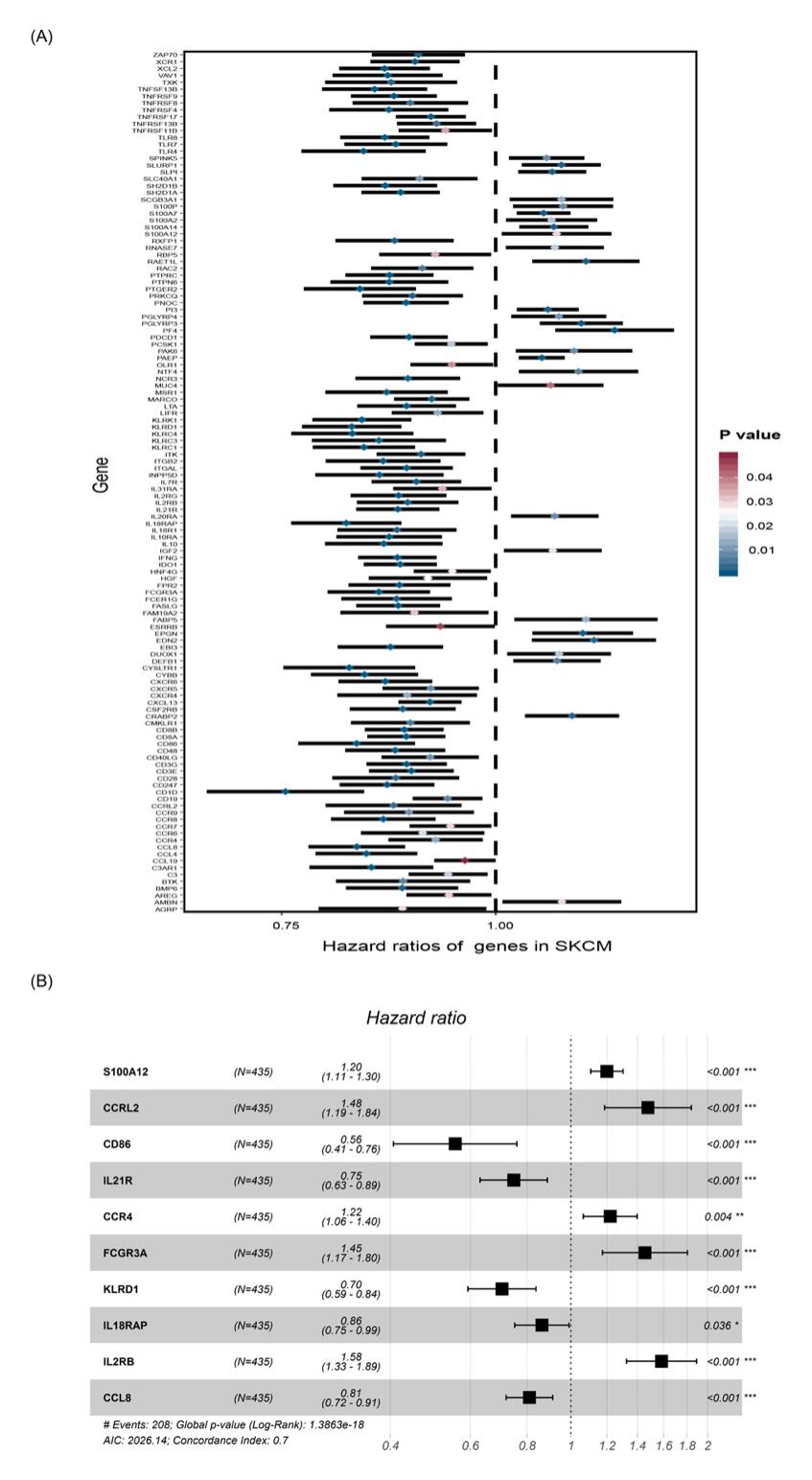
 **Supplementary Figure 1. (A)** Univariate Cox regression analysis of 125 candidate immune genes. **(B)** A forest map for the 10-gene signature constructed through multivariate Cox regression analysis.

**Supplementary Figure 2. (A)** Exploration of the association between clinicopathological characteristics and prognostic signature with the Chi-square test. Kaplan-Meier analysis of survival rates among BARF^wt^ **(B)**, BARF^mut^ **(C)**, NRAS^wt^ **(D)**, NRAS^mut^ **(E)**, Stage I+II **(F)**, and Stage III+IV **(G).**


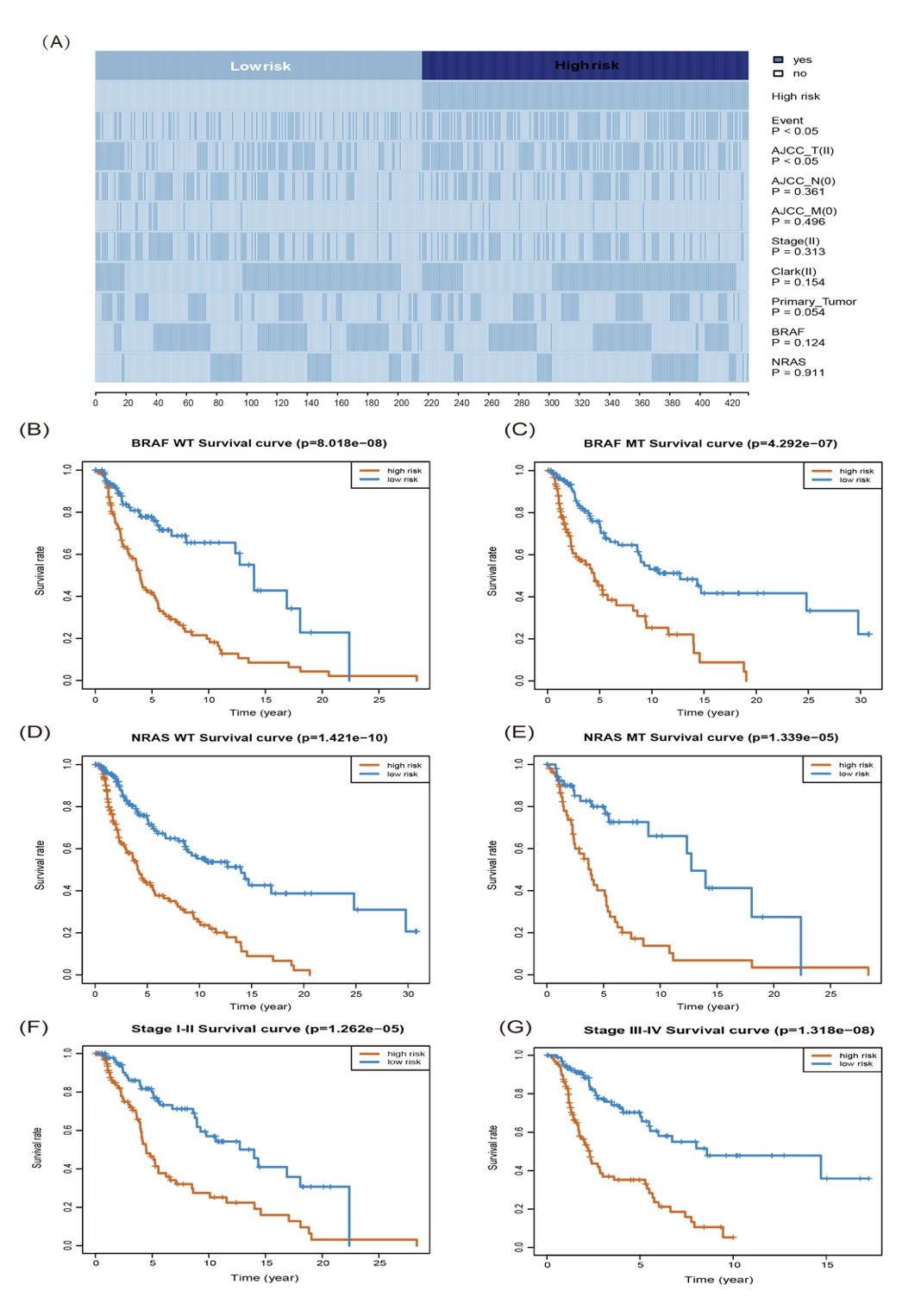


**
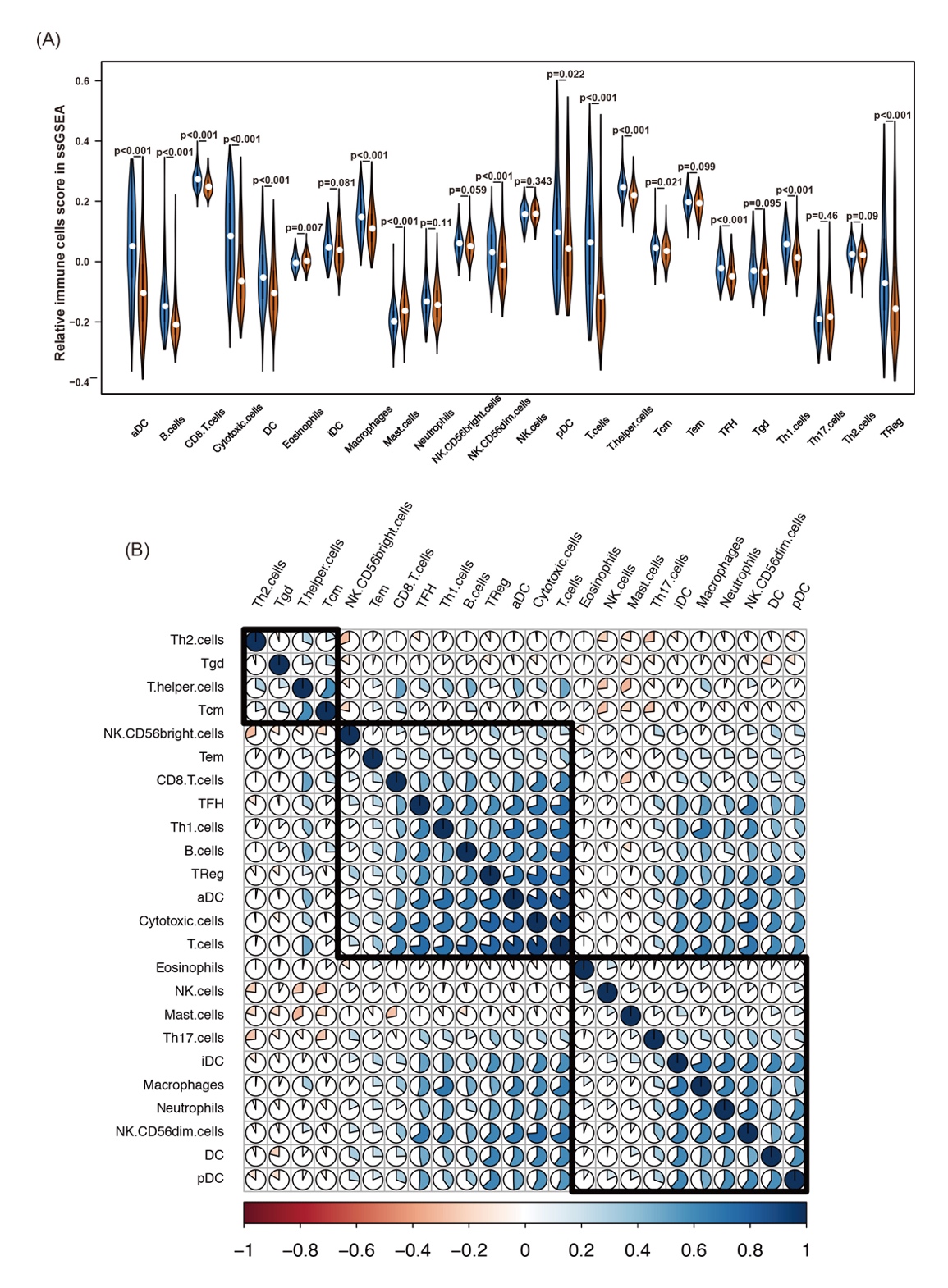
Supplementary Figure 3. (A)** Association of 24 immune cells infiltration abundances and risk scores. The blue and red violins represent the 10-IRG signature low- and high-risk groups, respectively. The white points inside the violin represent median values. **(B)** Heatmap of pair-wise correlation between 24 immune cell populations. According to the interaction between cells, three cell modules were defined.

**Supplementary Figure 4. (A)** Infiltration levels of 24 immune cells in melanoma patients from the GEO cohort (GSE19234). Association of risk score with age, gender, stage, and cluster was estimated based on gene expression using the ssGSEA method. **(B)** Kaplan-Meier curve evaluating the survival rate of melanoma patients in GSE19234 (P = 2.516e-01). **(C)** Difference in 24 immune cells infiltration abundances between two risk groups. The blue and red violins represent the 10-IRG signature low- and high-risk group, respectively. The white points inside the violin represent median values.


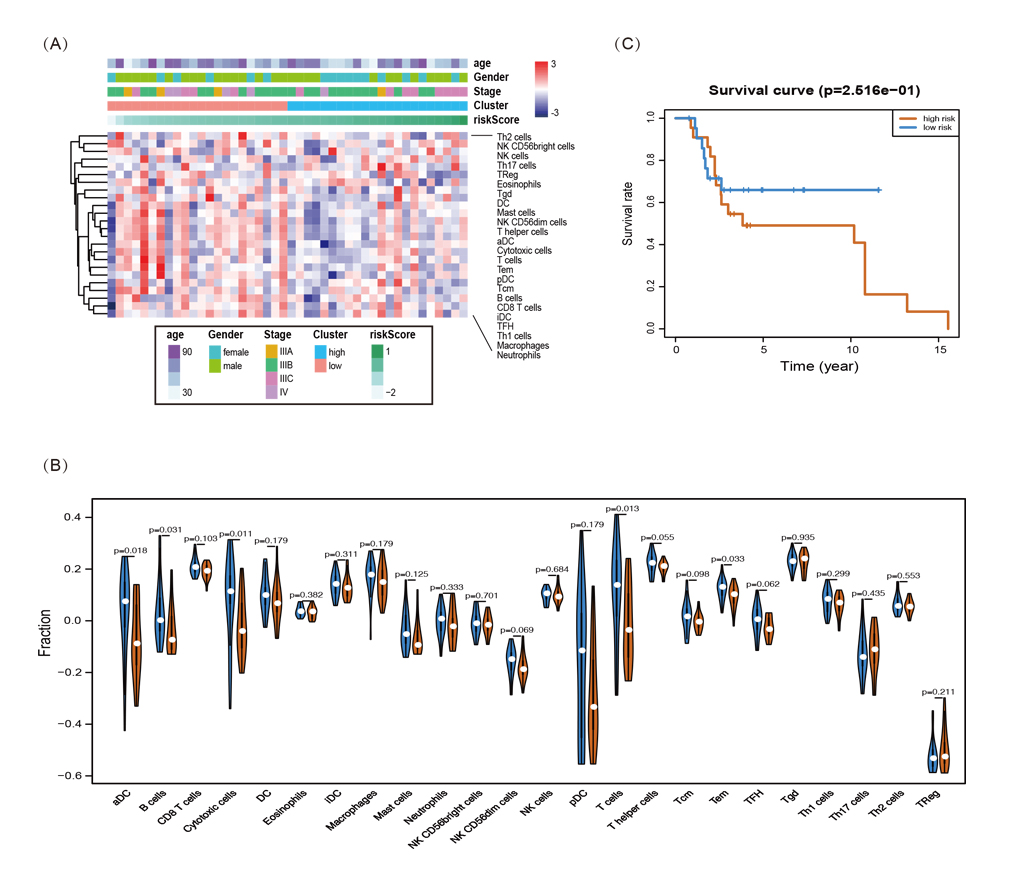


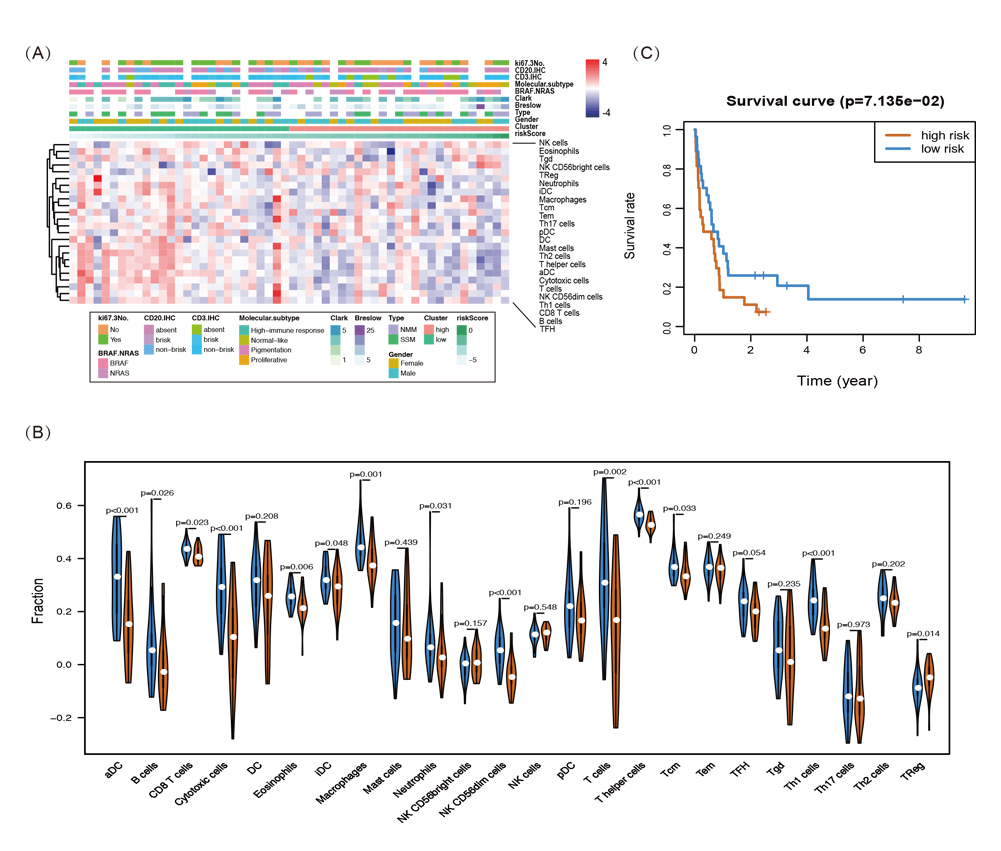
 **Supplementary Figure 5. (A)** Infiltration levels of 24 immune cells in melanoma patients from the GEO cohort (GSE22153). Association of risk score with diverse variables was estimated based on gene expression using the ssGSEA method. **(B)** Kaplan-Meier curve evaluating the survival rate of melanoma patients in GSE22153 (P = 7.135e-02). **(C)** The difference in 24 immune cells infiltration abundances between two risk groups. The blue and red violins represent the 10-IRG signature low- and high-risk groups, respectively. The white points inside the violin represent median values.


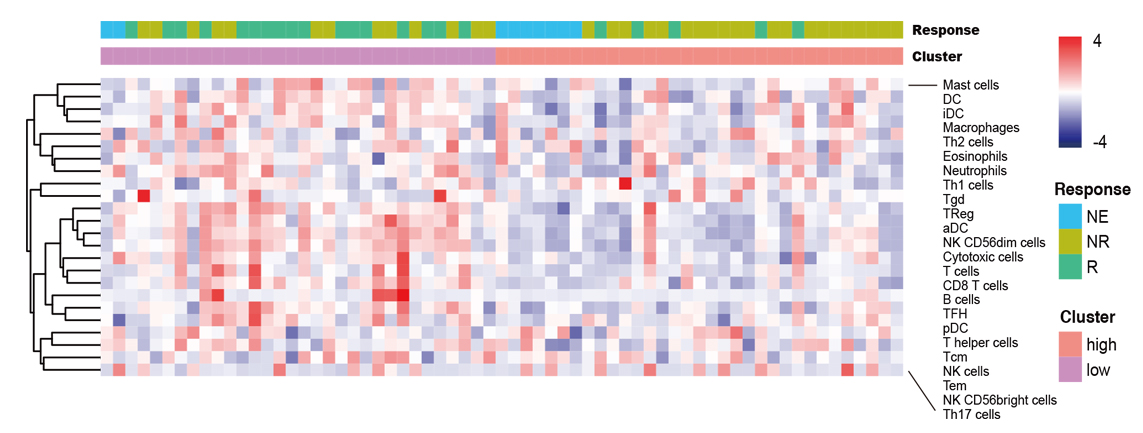
 **Supplementary Figure 6.** Infiltration levels of 24 immune cells in the microenvironment of melanoma patients in the GEO cohort (GSE35640). Association of risk score with response to immunotherapy was estimated based on gene expression using the ssGSEA method.

**Supplementary Figure 7**. A flowchart of the study.


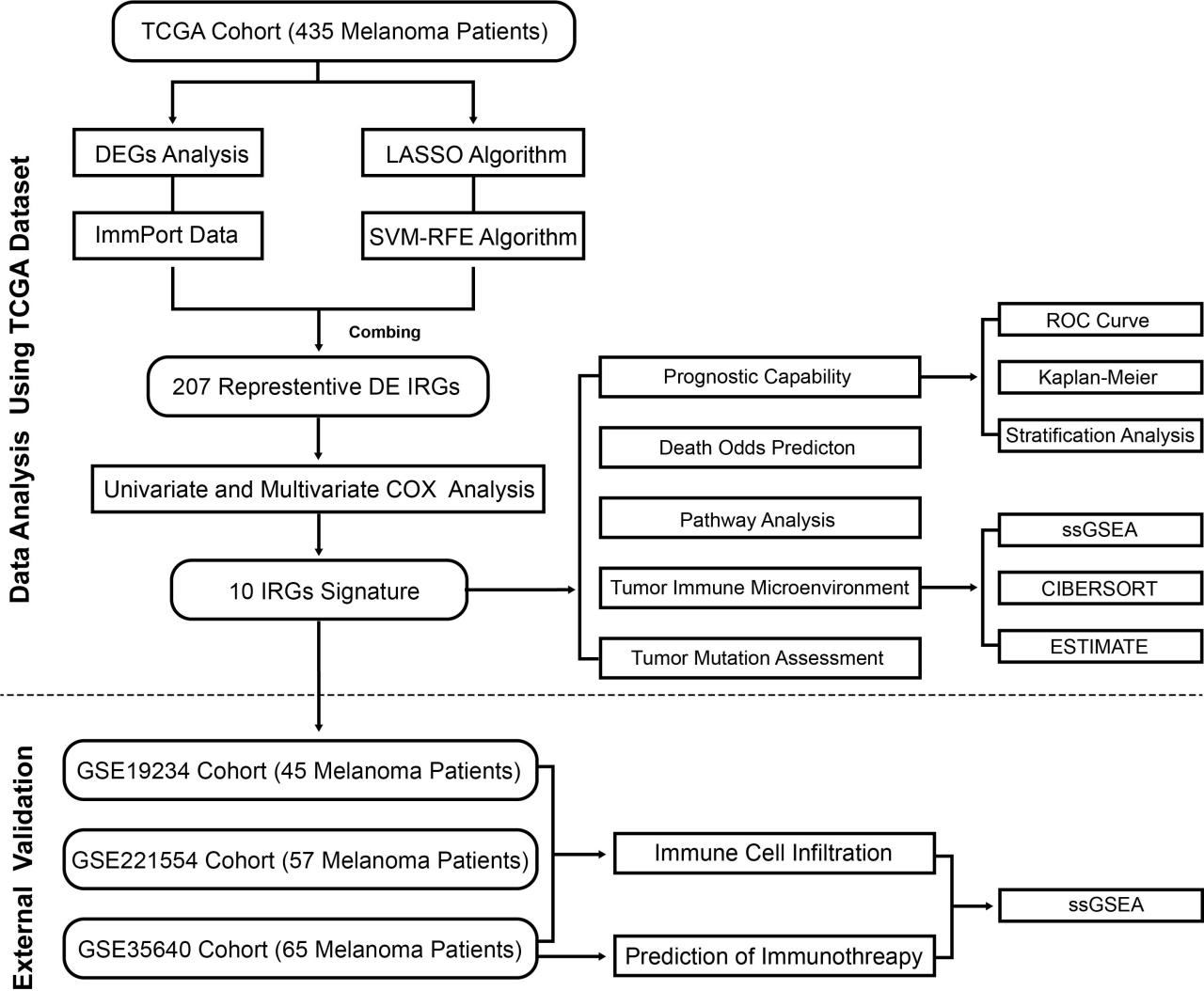

Supplement: Supplementary file 1 [file Data_Sheet_1.docx]
